# Preparation and catalytic activity of bone-char ash decorated with MgO - FeNO_3_ for ozonation of reactive black 5 dye from aqueous solution: Taguchi optimization data

**DOI:** 10.1016/j.dib.2017.05.025

**Published:** 2017-05-19

**Authors:** Ghorban Asgari, Somaye Akbari, Abdol Motaleb Seid Mohammadi, Ali Poormohammadi, Bahman Ramavandi

**Affiliations:** a Social Determinants of Health Research Center (SDHRC), Department of Environmental Health Engineering, Hamadan University of Medical Sciences, Hamadan, Iran; b Department of Environmental Health Engineering, Hamadan University of Medical Sciences, Hamadan, Iran; c Environmental Health Engineering Department, Faculty of Health and Nutrition, Bushehr University of Medical Sciences, Bushehr, Iran

**Keywords:** Reactive black 5, MgO- FeNO_3_, Bone-char ash, Catalytic ozonation, Taguchi, Optimization

## Abstract

Reactive dye is one of the most applicable dyes in textile industries which its release to the water bodies creates a concern for environmentalists. Here, in this data article a bone-char (BC) ash decorated with MgO-FeNO_3_ for removing reactive black 5 (RB5) dye in a catalytic ozonation process (COP) system. Operational parameters data such as initial RB5 concentration, pH, catalyst dosage, and reaction time were optimized using Taguchi method. The optimal conditions for initial RB5 concentration, pH, catalyst dosage, and reaction time were determined 10 mg/L, 10, 0.1 g/L, and 15 min, respectively. Data of Taguchi optimization tests indicated that the initial RB5 concentration had a significant influence on RB5 removal efficiency (54.03%) during the catalytic ozonation process, and reaction time had lower contribution (2.04%).

**Specifications Table**

**Table:** 

Subject area	*Environmental engineering*
More specific subject area	*Environmental technology*
Type of data	*Table and figure*
How data was acquired	*All tests were conducted in a glass reactor, in the presence of various dosages of BC ash decorated with MgO-FeNO*_*3*_*catalyst.*
	*The concentrations of RB5 samples were measured using a UV-visible spectrophotometer (DR5000) at 597* *nm wavelength.*
Data format	*Analyzed*
Experimental factors	*Monitoring RB5 concentrations under various levels of initial RB5 concentration, pH, catalyst dosage, and reaction time for achieving the optimum removal conditions of RB5 from aqueous samples using BC ash decorated with MgO-FeNO*_*3*_*as catalyst.*
Experimental features	*Treatment of RB5 using BC ash decorated with MgO-FeNO*_*3*_*catalyst as an advanced catalytic oxidation process*
Data source location	*Chemistry laboratory of water and wastewater, Hamadan University of Medical Sciences, Iran.*
Data accessibility	*Data are presented in the article*

**Value of the data**

• This data article presents a facile statistical method to optimize RB5 removal from aqueous solution using catalytic ozonation process with modified BC ash-MgO-FeNO_3_ as an eco-friendly process.
• The data article focused on the synthesis of new cost-benefit catalyst, and its application for removing organic dyes from aqueous solution.
• This dataset can be also used for reducing of other reactive dyes from textile wastewaters which are challenging pollutants for the water bodies.

## 1 Data

**Fig. 1:**
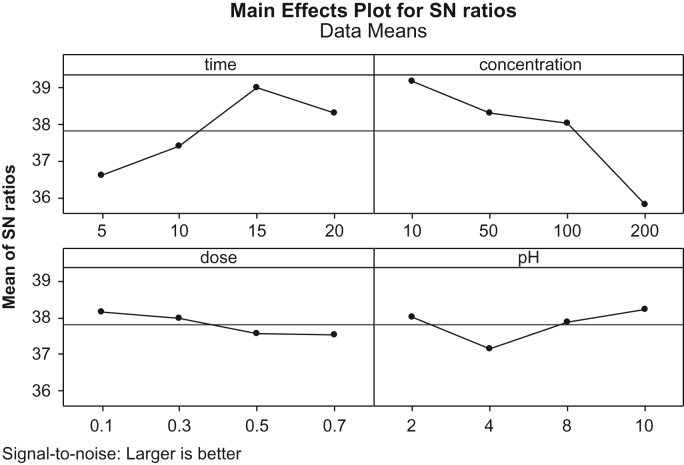
The effect of factors on *S*/*N* ratio in RB5 removal.

**Fig. 2:**
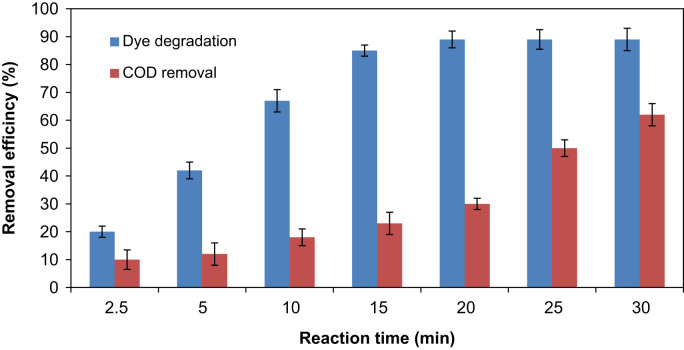
The effect of catalytic ozonation process in removal of RB5 and COD under optimum conditions (initial RB5 concentration: 10 mg/L, pH: 10, catalyst dose: 0.1 g/L).

This brief article describes the use of new synthesized catalytic for removing a dye from synthetic wastewater and optimizing the process using Taguchi method. Table 1 presents the studied parameters and their ranges. In Table 2, we presented the signal-to-noise (*S*/*N*) ratio of each experiment from different arrangement (*S*/*N* ratio is a factor that is used for evaluating the experimental data). Table 3 illustrates the mean of the *S*/*N* ratio (*M*_S/N_) of each factor at a certain level. Fig. 1 shows the effect of each studied parameters on the *S*/*N* ratio. Fractional sum of squares and percentage contribution of each factors on the catalytic ozonation process efficiency in RB5 removal are illustrated in Table 4. Kinetic data are demonstrated in Table 5. Eventually, the process efficiency in removal of COD and RB5 was studied and the findings are depicted in Fig. 2.

**Table 1:** Controllable factors and their levels.

Designation	Explanation	Level 1	Level 2	Level 3	Level 4
A	Reaction time (min)	5	10	15	20
B	Initial dye concentration (mg/L)	10	50	100	200
C	pH	2	4	8	10
D	Catalyst dose (g/L)	0.1	0.3	0.5	0.7

**Table 2:** The *S*/*N* ratio of each experiment from different arrangement of factors.

Run	*A*	*B*	*C*	*D*	MAE%	MAE%	*S*/*N*
1	5	10	2	0.1	91	93	36/57967
2	5	50	4	0.3	92	80	37/387245
3	5	100	8	0.5	77	76	**38**/**9919**
4	5	200	10	0.7	80	59	38/3054025
5	10	10	10	0.3	90	93	**39**/**1523**
6	10	50	8	0.1	94	89	38/3101325
7	10	100	4	0.7	90	85	38/0113025
8	10	200	2	0.5	62	59	35/79052
9	15	10	4	0.5	91	92	**38**/**1691**
10	15	50	2	0.7	94	93	37/9854675
11	15	100	10	0.1	75	83	37/5722625
12	15	200	8	0.3	87	88	37/5374375
13	20	10	8	0.7	91	92	38/0086
5	20	50	10	0.5	97	98	37/13771
15	20	100	2	0.3	86	92	37/8737125
16	20	200	4	0.1	80	88	**38**/**0102**

**Table 3:** Response table for *M*_S/N_ ratios for the tested factors and corresponding levels.

Level	*A*	*B*	*C*	*D*
1	36/57967	39/1523	38/0086	38/1691
2	37/38725	38/3101	37/1377	37/9854
3	38/9919	38/0113	37/8737	37/5722
4	38/3054	35/7905	38/0102	37/5374

**Table 4:** Fractional sum of squares and percentage contribution of each factors on RB5 removal.

Factors	DF	Seq SS	Adj SS	Adj MS	R%
Reaction time (min)	3	13.338	13.338	4.4461	2.54
Initial dye concentration (mg/L)	3	24.682	24.682	8.2273	54.04
pH	3	2.722	2.722	0.9072	5.95
Catalyst dose (g/L)	3	1.161	1.161	0.3872	16.32

**Table 5:** Kinetics of dye degradation at optimum conditions in catalytic ozonation process (COP) and single ozonation process (SOP).

**Parameters**	
***K***_**COP**_**, min**^**−1**^	***K***_**SOP**_**, min**^**−1**^
0.8	0.12

## 2 Experimental design, materials and methods

### 2.1 Synthesis of catalytic

The cow bone was put in an electric furnace at 800 °C during 2 h to produce BC ash. Then, BC ash was powdered using an electric mill and sieved with American Standard Test Sieve Series (ASTM) in the range 8–16 mesh. The obtained BC ash was modified using MgCl_2._ In order to modify the ash, 5 g of its powder was mixed with 1 M of MgCl_2_ and 1 N of potassium at 120 rpm, and then, it was dried at 60 °C for about 24 h. The dried product was cooled by a desiccator, and then heated in the electric furnace at 500 °C for 2 h. The produced BC was treated with 0.1 M of Fe(NO_3_)_6_.H_2_O at 500 °C for 2 h. Finally, it was denoted as BC-MgO- FeNO_3_ [1], [2].

### 2.2 Designation and optimization of COP experiments

Minitab 16 Statistical software was used for designing of 4 key parameters: initial RB5 concentration, pH, catalyst dose, and reaction time. These parameters were taken into account in the design of experiments based on the Taguchi method. Each parameter was configured at 4 levels (see Table 1). All experiments were run in duplicate.

The signal-to-noise (*S*/*N*) ratio was applied to evaluate the experimental data. Among three obtained values of *S*/*N* ratio, the highest value was selected as optimum condition. In this regards, all related equations are described and presented in our previous study [3].

To conclude the optimum conditions for the RB5 removal experiments, relationship between each parameter and the percentage (%) contribution on the dye removal, the analysis of mean (ANOM) and analysis of variance (ANOVA) were used.

Primarily, the mean of the *S*/*N* ratio (*M*_S/N_) of each factor at a certain level determined [3]. Next, the higher *M*_S/N_ as better characteristics was selected as optimum conditions of each parameter. The influence percentage of each factor on RB5 removal efficiency during the catalytic ozonation process was found from substituting the factorial sum of squares (SS_F_), the total sum of square SS_T_ and the variance of error (*V*_E_) (according to our previous study [3]).

To demonstrate the effect of BC-MgO- FeNO_3_ on dye degradation, the kinetic of day degradation was studied. The $k_{o-overall}^{dye}$ parameter as the pseudo-first-order rate constants ($\min^{-1}$) was determined using the following Equation:

(1) $$\ln\;\frac{C_{{\left(dye\right)}_{t}}}{C_{\left(dye\right)t=0}}=-k_{o-overall}^{dye}t$$

The acquired data from both COP and sole ozonation process (SOP) well fitted the pseudo-first-order kinetic. *k*_sop_ was also calculated by the equation described elsewhere [1].

### 2.3 Analytical methods

The concentrations of RB5 in reaction samples were measured using a UV-visible spectrophotometer (DR5000) at 597 nm wavelength [4]. In order to determine pH_zpc_ (pH of zero point of charge) 0.01 M of sodium chloride solution as the electrolyte prepared and HCl or NaOH (0.1 N) were used to adjust pH of the solutions. To investigate the COP efficiency in RB5 mineralization, COD removal was also measured according to Standard Method of potassium dichromate oxidation) [5], [6], [7].
